# Clinical improvement of renal amyloidosis in a patient with systemic-onset juvenile idiopathic arthritis who received tocilizumab treatment: a case report and literature review

**DOI:** 10.1186/s12882-017-0573-y

**Published:** 2017-05-12

**Authors:** Songkiat Chantarogh, Soamarat Vilaiyuk, Thipwimol Tim-Aroon, Suchin Worawichawong

**Affiliations:** 1 Division of Nephrology, Faculty of Medicine Ramathibodi Hospital, Mahidol University, Postal address: 270, Rama 6 Road, Phayathai, Ratchathewi, Bangkok, 10400 Thailand; 2 Division of Rheumatology, Faculty of Medicine Ramathibodi Hospital, Mahidol University, Postal address: 270, Rama 6 Road, Phayathai, Ratchathewi, Bangkok, 10400 Thailand; 3 Division of Medical Genetics, Department of Pediatrics, Faculty of Medicine Ramathibodi Hospital, Mahidol University, Postal address: 270, Rama 6 Road, Phayathai, Ratchathewi, Bangkok, 10400 Thailand; 4 Department of Pathology, Faculty of Medicine Ramathibodi Hospital, Mahidol University, Postal address: 270, Rama 6 Road, Phayathai, Ratchathewi, Bangkok, 10400 Thailand

**Keywords:** Renal amyloidosis, Systemic-onset juvenile idiopathic arthritis, Tocilizumab

## Abstract

**Background:**

Juvenile idiopathic arthritis (JIA) is a common rheumatic disease in children and adolescents. Although JIA may cause secondary amyloidosis, this is a rare complication in patients with JIA and other rheumatic diseases. Many previous studies have revealed that common heterozygous or homozygous mutations in the *MEFV* gene are associated with systemic-onset JIA (SJIA).

**Case presentation:**

We herein report a case involving a 19-year-old female patient with difficult-to-control SJIA. She developed progressive proteinuria without clinical signs or symptoms of edema. Renal amyloidosis was diagnosed by renal pathologic examination, which demonstrated deposition of eosinophilic amorphous material in the interlobular arteries, arterioles, and interstitium. Electron microscopy showed fibrillary material deposits with a diameter of 8 to 10 nm. A heterozygous E148Q mutation in the *MEFV* gene was identified. Conventional disease-modifying anti-rheumatic drugs and etanercept had been used to treat the SJIA, but the disease could not be controlled. Therefore, we decided to start tocilizumab to control the disease activity. However, the patient was unable to receive a standard dose of tocilizumab in the early period of treatment because of socioeconomic limitations. Her disease course was still active, and proteinuria was found. Therefore, tocilizumab was increased to a dose of 8 mg/kg every 2 weeks (standard dose of SJIA), and the patient exhibited a clinical response within 3 months.

**Conclusion:**

Refractory SJIA associated with renal amyloidosis is an uncommon cause of proteinuria in adolescents. Tocilizumab may be a beneficial treatment for renal amyloidosis in patients with SJIA.

## Background

Amyloidosis is a group of systemic or localized diseases characterized by extracellular deposition of amyloid fibrils in multiple organs [1, 2]. Organ involvement depends on the subtype of amyloidosis. Secondary or reactive amyloidosis can develop in patients with chronic inflammatory diseases and affects multiple organs such as the kidneys, liver, heart, gastrointestinal system, and autonomic nervous system [3]. The amyloidogenic precursor protein in secondary amyloidosis is mainly synthesized by the hepatic system in a chronic inflammatory state. Accumulation of amyloid fibrils leads to progressive dysfunction of affected organs [4]. Juvenile idiopathic arthritis (JIA) is a common rheumatic disease in children and may cause secondary amyloidosis [5, 6]. However, amyloidosis is still a rare complication in patients with JIA and other rheumatic diseases [5, 6]. Many previous studies have revealed that common heterozygous or homozygous mutations in the *MEFV* gene are associated with systemic-onset JIA (SJIA) [7–9]. We herein report a case involving an adolescent female patient who was diagnosed with SJIA and developed progressive proteinuria. She was found to carry a heterozygous E148Q mutation in the *MEFV* gene. Renal pathological examination confirmed the diagnosis of amyloidosis. This case will help general practitioners and pediatricians to recognize the etiology of proteinuria in adolescents with JIA.

## Case presentation

### Clinical history and investigation results

A 19-year-old female Thai patient had been diagnosed with SJIA 10 years previously and had been developing progressive proteinuria for 1 year. Her initial manifestations of SJIA were quotidian fever, polyarthritis, and pericardial effusion. Although she received pulse methylprednisolone, high-dose prednisolone, and multiple disease-modifying anti-rheumatic drugs from her previous hospital (including methotrexate, sulfasalazine, and cyclosporine A), her disease activity remained mostly active. At the age of 13 years, she was referred to Ramathibodi Hospital for proper management by a pediatric rheumatologist. Her blood samples were negative for antinuclear antibody, anti-double-stranded DNA, and HLA-B27 with normal levels of C3 (1550 μg/mL; reference range, 900–1800 μg/mL) and C4 (551 μg/mL; reference range, 100–600 μg/mL). No underlying disease was found in her family members. During the first year of follow-up at Ramathibodi Hospital, the patient was treated with 25 mg/week of etanercept [an anti-tumor necrosis factor (TNF) agent], 25 mg/week of methotrexate, 2 g/day of sulfasalazine, and 5 mg/day of prednisolone. After 6 months of treatment with etanercept, she still had severe polyarthritis. Therefore, the etanercept was discontinued, and tocilizumab, a humanized anti-interleukin (IL)-6 receptor antibody, was started at that time. However, we could not use a standard dose of tocilizumab for SJIA (8 mg/kg every 2 weeks) in the early treatment period because of the patient’s socioeconomic situation. Therefore, she received tocilizumab at a dose of 8 mg/kg every 4 weeks. She partially responded to the tocilizumab; her IL-6 level slightly declined from 1105.0 to 574.2 pg/mL 5 months after starting the treatment. Her disease course still waxed and waned. Her arthritis relapsed while receiving the tocilizumab. Therefore, pulse methylprednisolone at 1 g/month, leflunomide at 20 mg/dose, and hydroxychloroquine at 200 mg/day were gradually added to the tocilizumab regimen. The rheumatologist noticed her first episode of albuminuria when her urine albumin dipstick result was 2+. This proteinuria showed evidence of progression at her 1-year follow-up, when her urinary protein-to-creatinine ratio (UPCR) exhibited deterioration from 0.87 to 3.00 (normal ratio, <0.2). A nephrologist was then consulted to diagnose the cause of the progressive proteinuria on the background of refractory SJIA.

On physical examination, her vital signs were normal. She was cachectic (body weight, <3rd percentile) and had a short stature (height, <10th percentile). Her wrists, knees, and ankles were stiff and inflamed. There were no signs or symptoms of edema. Other physical findings were unremarkable. Her urinalysis showed an inactive sediment. Significant proteinuria was confirmed by a 24-h urine collection method, which showed a total urine protein of 1295 mg/day (1792 mg/1.73m^2^/day). Blood chemistry analysis showed hypoalbuminemia (2.68 g/dL), a normal serum cholesterol level (152 mg/dL), and a normal serum creatinine level (0.47 mg/dL). Infectious screenings were negative for hepatitis B, hepatitis C, and human immunodeficiency viral infection. Other diagnostic investigation results are listed in Table 1.

**Table 1:** Clinical characteristics and laboratory results

Parameters	Before amyloidosis treatment	At 1-year follow-up
Clinical characteristics		
- Number of joints involved	19	11
- CHAQ-DI score	1.37	1.25
Immunology		
- IL-6, pg/mL	624.5 (0–7)	528.7 (0–7)
- ESR, mm/h	72 (5–20)	10 (5–20)
- CRP, mg/L	76.23 (<5)	3.40 (<5)

Data are presented as the laboratory result with reference range in parentheses unless otherwise indicated

*CHAQ-DI* Childhood Health Assessment Questionnaire Disability Index, *IL-6* interleukin-6, *ESR* erythrocyte sedimentation rate, *CRP* C-reactive protein

### Renal biopsy findings

**Fig. 1:**
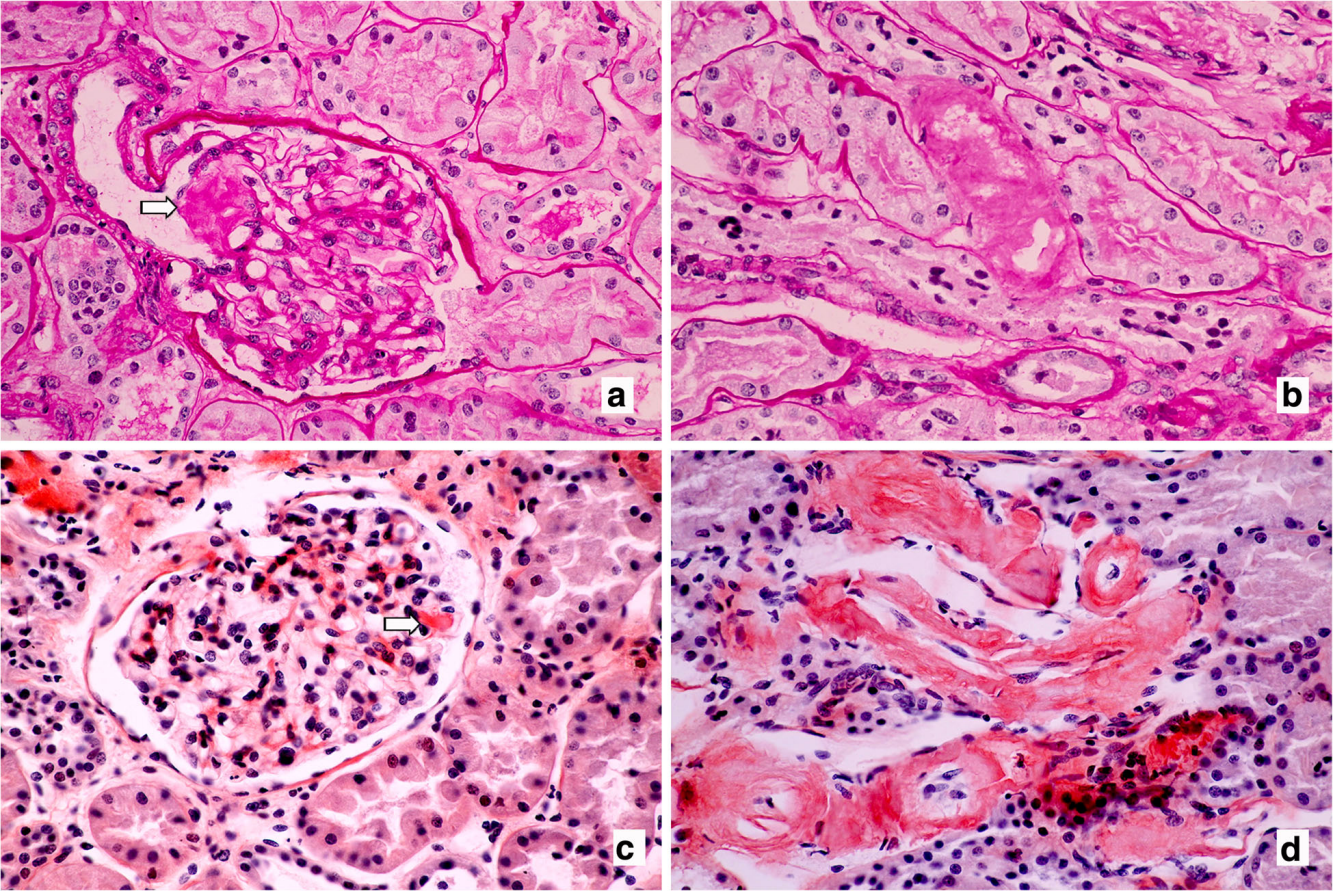
Depositions of amorphous eosinophilic material (*arrow*) in the (**a**) glomerular hilum and (**b**) arteriolar wall (×400, periodic acid–Schiff). Positive Congo red staining in the (**c**) mesangium and (**d**) arteriolar wall and interstitium (×400, Congo red)

**Fig. 2:**
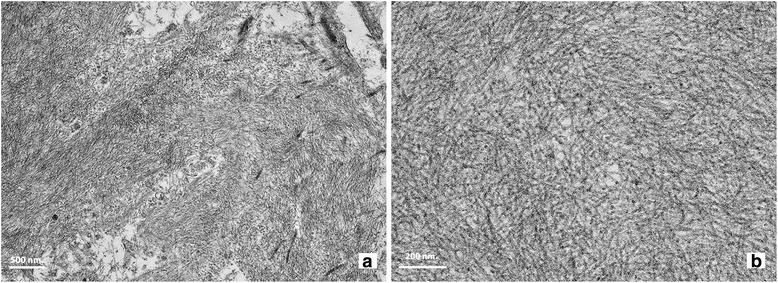
Electron micrograph shows (**a**) fibrillary deposits in the arteriolar wall and interstitium (×12,000). **b** Randomly oriented fibrils measuring 8 to 10 nm in diameter (×40,000)

The renal tissue contained eight nonsclerotic glomeruli. The glomeruli were unremarkable. The mesangium showed focal expansion without hypercellularity. Depositions of acellular eosinophilic amorphous material were seen in the glomerular hilum, mesangium, arteriolar wall, and interstitium (Fig. 1a, b). The material demonstrated fuchsinophilic staining, and Congo red staining was positive (Fig. 1c, d) with apple green birefringence under polarized microscopy. Mild tubular atrophy and interstitial fibrosis were also seen. An immunofluorescence study was negative for IgG, IgM, IgA, C3, C1q, fibrinogen, kappa, and lambda. Electron microscopy revealed randomly oriented fibrils of 8 to 10 nm in diameter in the mesangium, interstitium, and arteries (Fig. 2a, b). The glomerular basement membrane was unremarkable, but the podocytes showed partial foot process effacement under electron microscopy. These biopsy findings confirmed renal amyloidosis.

### Clinical follow-up after renal biopsy

Because the amyloidosis was secondary to uncontrolled SJIA, the tocilizumab was increased from a dose of 8 mg/kg every 4 weeks to a dose of 8 mg/kg every 2 weeks. We also started enalapril at 5 mg/day (0.12 mg/kg/day) for an additional antiproteinuric effect. The other drugs were continued at their same dosages. Two months later, the patient’s Childhood Health Assessment Questionnaire Disability Index score decreased. Her UPCR had also decreased from 3.00 to 0.92, and her arthritis improved 3 months after the tocilizumab increment. At the 1-year follow-up, her UPCR had decreased to 0.23 and renal function remained stable, with a serum creatinine concentration of around 0.42 to 0.52 mg/dL. Her C-reactive protein concentration had returned to normal and her IL-6 concentration had slightly decreased. Other follow-up investigation results are listed in Table 1.

### *MEFV* mutation analysis

A 3-mL venous blood sample was collected from the patient for DNA extraction. Next-generation sequencing was performed by SureSelect V5 using the Illumina HiSeq 4000 platform. Sanger sequencing was performed for variant verification. Variants of the *MEFV* gene (NM#000243, transcript ID: ENST00000219596) were analyzed. A heterozygous c.442G > C (pE148Q) mutation in the *MEFV* gene was identified.

## Discussion

Renal amyloidosis is a rare cause of proteinuria in children. The prevalence of amyloidosis is higher in patients with JIA than in the general pediatric population [10, 11], and among the seven subtypes of JIA, SJIA is associated with the highest prevalence of amyloidosis [10, 12]. In the present case, amyloidosis occurred 10 years after the onset of SJIA, similar to a previous study [12]. In many types of amyloidosis, the kidneys are the predominant organ involved. Proteinuria is the most common clinical presentation [5].

The *MEFV* gene encodes a protein called pyrin or marenostrin, which inhibits the processing of IL-1β to an active form through the regulation of nuclear factor-KB and caspase-1 [9]. Pyrin deficiency results in uncontrolled production of active IL-1β [13]. The *MEFV* gene is also responsible for familial Mediterranean fever, which is an autosomal recessive disorder [13]. Multiple mutations (polymorphisms) of the *MEFV* gene in one allele have been reported in patients with SJIA [7–9, 14]. The present patient only had a heterozygous E148Q mutation in the *MEFV* gene, which is a common polymorphism. Whether the E148Q polymorphism is a benign or disease-causing mutation remains controversial. Although glutamic acid has been conserved at position 148 throughout evolution, thus favoring mutation, the general populations of many ethnicities exhibit a high frequency of E148Q (Egyptian, 6.66%; Jewish, 4.30%–6.60%; Greek, 1.30%; Japanese, 23.70% [9, 14–16]; and Thai, 24.3% [unpublished data]). Without other polymorphisms in the *MEFV* gene, the presentation of amyloidosis of our patient may or may not have been related to the heterozygous E148Q mutation. Further functional studies with large samples from both the general population and patients with SJIA are needed to clarify the penetrance of E148Q.

Although we cannot classify the specific subtype of the amyloidogenic precursor in our country, AA amyloidosis is the most common subtype in patients with various chronic inflammatory diseases, including SJIA [3–6]. Therefore, we treated the patient in this case based on current evidence regarding treatment of AA amyloidosis. The main principle of management is suppression of the inflammatory process and control of the underlying disease [5, 17]. The clinical characteristics and treatment of JIA-related renal amyloidosis are shown in Table 2. Three of six patients were diagnosed with SJIA, and two responded to chlorambucil or leflunomide treatment for renal amyloidosis [18, 19]. Cantarini et al. [8] could not demonstrate the efficacy of colchicine in one patient with SJIA because of loss to follow-up. Some studies have shown the efficacy of chlorambucil for JIA-related amyloidosis [10, 11]. One case-cohort study [10] revealed that 80% of patients receiving chlorambucil achieved 10-year survival compared with only 24% of patients who had never received cytostatic treatment. However, there is lack of supporting evidence from randomized controlled trials (RCTs) regarding the efficacy of chlorambucil and leflunomide in patients with JIA-related amyloidosis.

**Table 2:** Clinical characteristics and treatment of reported patients with secondary amyloidosis related to juvenile idiopathic arthritis

Reference	Age (y)/Sex	Duration of JIA before diagnosis of amyloidosis (y)	Systemic features of JIA^a^	ESR or CRP, Initial to last FU (mm/h or mg/dL)	Organ involvement of amyloidosis	eGFR, initial to last FU (mL/1.73 m^2^/min)	Proteinuria, Initial to last FU (mg/day)	Treatment before diagnosis of amyloidosis	Additive treatment after diagnosis of amyloidosis	Duration of FU after diagnosis of amyloidosis	Efficacy of amyloidosis treatment
Duarte et al. 2005 [18]	9/F	5.5	Yes	ESR 125 to 27	Renal	NA	UPCR (mg/mmol) 1057 to 47	MTX, CSA, GCs, and ETA	CRB	14 months	Yes
Okuda et al. 2006 [30]	26/F	12	No	CRP 15 to 0.03	GI, Renal	66 to 124	700 to normal	ATM, BCL, D-PEN, SFZ, MTX, and GCs	TCZ	42 months	Yes
Nowak et al. 2009 [31]	31/F	10	No	ESR 40 to 26	Renal	38.6 to 140	5600 to <1100	CRB, GCs, and various DMARDs	ADA	20 weeks	Yes
Cantarini et al. 2009 [8]	9/F	2	Yes	CRP 14.7 to NA	Renal	NA	18,130 to NA	MTX, CSA, and GCs	COL, ↑ dose of MTX	NA (lost to FU)	NA (lost to FU)
Hakala et al. 2013 [32]	29/F	23	NA	CRP 64 to normal	Renal	27 to 51	1300 to normal	CYC, GCs, and various DMARDs	ADA, ETA, INF, ANA, ABA, TCZ	24 months	Yes
Saha et al. 2013 [19]	12/F	3	Yes	NA	Renal	128 to 128	UPCR (mg/g) 12.5 to 1.5	MTX, HCQ, and GCs	LFN	18 months	Yes

^a^Fever, hepatosplenomegaly, lymphadenopathy, and serositis

*F* female, *JIA* juvenile idiopathic arthritis, *ESR* erythrocyte sedimentation rate, *CRP* C-reactive protein, *eGFR* estimated glomerular filtration rate, *FU* follow-up, *NA* not available, *UPCR* urine protein-to-creatinine ratio, *MTX* methotrexate, *CSA* cyclosporine A, *GCs* glucocorticoids, *ETA* etanercept, *CRB* chlorambucil, *GI* gastrointestinal, *ATM* sodium aurothiomalate, *BCL* bucillamine, *D-PEN* D-penicillamine, *SFZ* sulfasalazine, *TCZ* tocilizumab, *DMARDs* disease-modifying anti-rheumatic drugs, *ADA* adalimumab, *COL* colchicine, *CYC* cyclophosphamide, *INF* infliximab, *ANA* anakinra, *ABA* abatacept, *HCQ* hydroxychloroquine, *LFN* leflunomide

Many studies have also demonstrated inadequate efficiency of anti-TNF-α medications for treatment of SJIA; remission was achieved in less than half of all patients in these studies, and relapsing disease was common [20–23]. Two studies have shown the efficacy of anti-TNF therapy in treating amyloidosis secondary to autoimmune diseases, including JIA [24, 25]. A retrospective study of 15 patients with amyloidosis with underlying rheumatic diseases revealed that 54.5% of patients receiving infliximab (81%) or etanercept (19%) showed renal improvement and 17% showed renal progression at the end of follow-up [25]. Two RCTs demonstrated the efficacy of tocilizumab for the treatment of refractory SJIA in patients without amyloidosis [26, 27]. Several retrospective studies have demonstrated clinical improvement rates of around 70% in adult patients with rheumatic disease with amyloidosis after tocilizumab treatment [28, 29]. However, patients with SJIA were not included in these studies; thus, the efficacy of tocilizumab treatment for amyloidosis secondary to SJIA remains unclear.

The present patient developed secondary amyloidosis despite receiving tocilizumab, which can be explained by her uncontrolled SJIA. The patient still had persistent active arthritis while receiving a tocilizumab dose of 8 mg/kg every 4 weeks. Although she had no systemic features, her inflammatory markers (IL-6 level of 624.5 pg/mL and C-reactive protein level of 76.2 mg/L) were persistently high, reflecting insufficient control of her disease activity. After the tocilizumab was increased from every 4 weeks to every 2 weeks, which is the same dosage reported in previous RCTs [26, 27], her clinical symptoms of arthritis were relieved and her inflammatory markers decreased within 3 months. She had stable renal function, and her UPCR had decreased to 0.23 at the 1-year follow-up. Therefore, tocilizumab is beneficial in treating amyloidosis secondary to SJIA when conventional therapies fail. However, the minimum dose of tocilizumab in such patients is 8 mg/kg every 2 weeks.

## Conclusion

We have described a case of renal amyloidosis secondary to refractory SJIA in a carrier of the E148Q mutation in the *MEFV* gene. Physicians should be aware of the possibility of amyloidosis development in patients with SJIA, especially when such patients show progressive proteinuria and uncontrolled disease. Early detection and appropriate treatment will affect clinical outcomes and lead to a better prognosis. Tocilizumab may be beneficial in patients with amyloidosis related to uncontrolled SJIA.

## Abbreviations

ABA: Abatacept
ADA: Adalimumab
ANA: Anakinra
ATM: sodium aurothiomalate
BCL: Bucillamine
CHAQ-DI: Childhood Health Assessment Questionnaire Disability Index
COL: Colchicine
CRB: Chlorambucil
CRP: C-reactive protein
CSA: Cyclosporine A
CYC: Cyclophosphamide
DMARDs: Disease-modifying antirheumatic drugs
D-PEN: D-penicillamine
eGFR: Estimated glomerular filtration rate
ESR: Erythrocyte sedimentation rate
ETA: Etanercept
FU: Follow-up
GCs: Glucocorticoids
GI: Gastrointestinal tract
HCQ: Hydroxychloroquine
IL: Interleukin
INF: Infliximab
JIA: Juvenile idiopathic arthritis
LFN: Leflunomide
MTX: Methotrexate
NA: Not available
RCT: Randomized controlled trial
SFZ: Sulfasalazine
SJIA: Systemic-onset juvenile idiopathic arthritis
TCZ: Tocilizumab
UPCR: Urine protein-to-creatinine ratio
